# A New Non-Pterodactyloid Pterosaur from the Late Jurassic of Southern Germany

**DOI:** 10.1371/journal.pone.0039312

**Published:** 2012-07-05

**Authors:** David W. E. Hone, Helmut Tischlinger, Eberhard Frey, Martin Röper

**Affiliations:** 1 Department of Biology and Environmental Sciences, University College Dublin, Dublin, Ireland; 2 Tannenweg 16, Stammham, Germany; 3 Staatliches Museum für Naturkunde Karlsruhe, Karlsruhe, Germany; 4 Museum Solnhofen, Bahnhofstraße 8, Solnhofen, Germany; College of the Holy Cross, United States of America

## Abstract

**Background:**

The ‘Solnhofen Limestone’ beds of the Southern Franconian Alb, Bavaria, southern Germany, have for centuries yielded important pterosaur specimens, most notably of the genera *Pterodactylus* and *Rhamphorhynchus*. Here we describe a new genus of non-pterodactyloid pterosaur based on an extremely well preserved fossil of a young juvenile: *Bellubrunnus rothgaengeri* (gen. et sp. nov.).

**Methodology/Principal Findings:**

The specimen was examined firsthand by all authors. Additional investigation and photography under UV light to reveal details of the bones not easily seen under normal lighting regimes was completed.

**Conclusions/Significance:**

This taxon heralds from a newly explored locality that is older than the classic Solnhofen beds. While similar to *Rhamphorhynchus*, the new taxon differs in the number of teeth, shape of the humerus and femur, and limb proportions. Unlike other derived non-pterodacytyloids, *Bellubrunnus* lacks elongate chevrons and zygapophyses in the tail, and unlike all other known pterosaurs, the wingtips are curved anteriorly, potentially giving it a unique flight profile.

## Introduction

Pterosaur diversity is currently in an exponential phase of discovery [1] with numerous new taxa being described each year. While many of these are from newly explored localities (e.g. the Middle Jurassic *Sericipterus* from Xinjiang, China [2]) or those major formations that are still being explored (such as the Jehol of China), the Late Jurassic Solnhofen Lithographic Limestone and some surrounding beds of “Solnhofen-type-lagerstätten” of the Southern Franconian Alb in Bavaria have been well studied. The first pterosaur specimen described in 1784 was recovered from the Solnhofen beds and since then hundreds of specimens have been recovered including those with superbly preserved soft tissues [3], [4]. Historically this material has formed much of the basis of pterosaur research for the first century of work on these flying reptiles. The Solnhofen genera *Pterodactylus* and *Rhamphorhynchus* were essential for taxonomic and evolutionary work on the dichotomy between the major groups of pterosaurs – the derived Pterodactyloidea and the basal paraphyletic assemblage of ‘rhamphorhynchoids’.

Important new pterosaur material continues to be uncovered in the Solnhofen and its surrounding beds (e.g. [5], [6]), but despite more than 200 years of collecting, few new putative taxa have been described in many decades. Indeed, much taxonomic revision has drastically reduced the number of genera and species known from these beds [7]–[10] such that currently around 12 species remain valid [11].

Here we report on a new non-pterodactyloid pterosaur based on a complete and articulated specimen of a young juvenile. Despite a close affinity with the well-known pterosaur *Rhamphorhynchus* (based on a large number of shared characteristics), *Bellubrunnus* gen. et sp. nov. is clearly separated from this and other rhamphorhynchid taxa by a number diagnostic of anatomical characters. Significantly, this specimen is from a locality that has only been explored in the last two decades and that predates the Solnhofen Limestone just as other Solnhofen-type lagerstätten of southern Franconia.

### Systematic Palaeontology

Pterosauria Kaup, 1834 [12].

Breviquartossa Unwin, 2003 [11].

Rhamphorhynchidae Seeley, 1870 [13].

*Bellubrunnus* gen. nov.

urn:lsid:zoobank.org:act:C309EFA2-8938-426F-9643-572225B53541.

*Bellubrunnus rothgaengeri* sp. nov.

urn:lsid:zoobank.org:act:C0C68EB9-D8A5-4C28-B517-4F14553B8119.

### Holotype

Specimen BSP–1993–XVIII–2 (BSP: Bayerische Staatssammlung für Paläontologie und historische Geologie, Munich, Germany) is a complete and articulated skeleton of a juvenile non-pterodactyloid pterosaur seen in ventral view.

### Etymology

From the Latin ‘bellus’ meaning beautiful and Brunn from the locality of the holotype specimen. This then is the beautiful one of Brunn. The species name honours Monika Rothgaenger who found the holotype.

### Definition and Diagnosis

Rhamphorhynchine pterosaur that differs from other rhamphorhynchines by the following diagnostic features: 22 or fewer teeth in total, no elongate zygopophyses or elongate chevrons in the tail, distal half of the humeral shaft straight, humerus significantly longer (1.4 times the length) of the femur, femoral head with no neck.

### Specimen History

**Figure 1:**
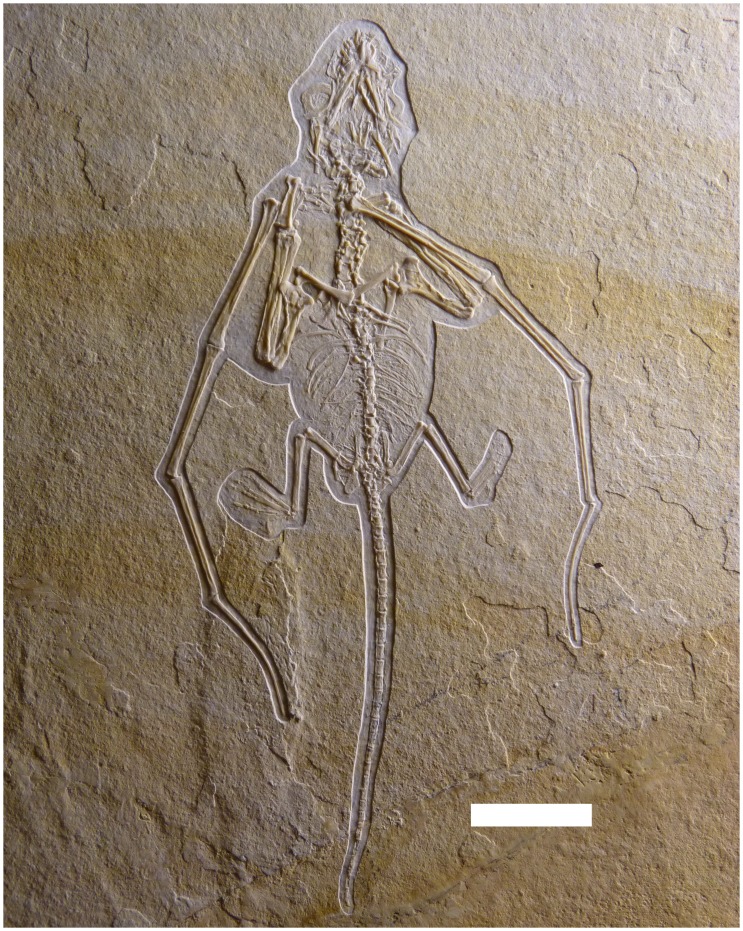
Holotype specimen of *Bellubrunnus* BSP XVIII–VFKO–A12. Scale bare 1 cm. *Full page width.*

The specimen was found in summer 2002 during an investigation of the Brunn quarry by Monika Rothgaenger, who was at the time in charge of the privately organised scientific excavation in cooperation with the Bavarian State Collection for Palaeontology, Munich and the Solnhofen Museum, Bavaria. The specimen was subsequently prepared by freelance preparator Martin Kapitzke in Stuttgart before coming to the Solnhofen Museum in 2003. While permanently housed in the Solnhofen Museum as specimen BSP–1993–XVIII–2 (formerly curated as BSP XVIII–VFKO–A12), the material is owned by Bavarian State Collection for Palaeontology and Geology, Munich, Bavaria, Germany (BSP). See Figure 1.

### Locality, Geological Setting and Stratigraphy

#### The fossil lagerstätte of brunn

The small village of Brunn is situated in Upper Palatinate, Eastern Bavaria, 25 km northwest of the city of Regensburg on the westernmost rim of the Southern Franconian Alb. The Brunn quarry is a small stone pit at the “Kohlstatt locality”, between the villages of Brunn and Wischenhofen, which was previously quarried for road building materials. Starting around 1990 some well-preserved fossils were discovered by collectors, and the first scientific excavations took place soon afterwards. These yielded many fossil plants and numerous invertebrate and vertebrate taxa. The Brunn quarry is now a protected site reserved for geological research only (Geological map of Bavaria 1∶25 000, sheet 6937, Laber and sheet 6837, Kallmünz).

#### Geological setting, age and stratigraphy

Palaeogeographically the Kohlstatt locality is part of the Plattenkalk deposits of the small Pfraundorf-Heitzenhofen Basin [14]. In contrast to the classical Solnhofen lithographic limestone in southern Franconia, the deposit was surrounded by active reefs, microbial bioherms and small coral reefs. The Rhytmic Plattenkalk of Brunn is of Late Kimmeridgian age and is dated within the lower part of the Beckeri-Zone, Subeumela Subzone [15]–[17]. Brunn is therefore among the stratigraphically oldest Fossillagerstätte of the Solnhofen type and is significantly older than the plattenkalk from Nusplingen in Baden-Württemberg, but younger than Wattendorf in Northern Franconia (Table 1). The section is some eight metres thick and consists of eight thin stacks of plattenkalk of up to 0.6 m thickness each, interrupted by layers of reworked plattenkalk. In the lower part of the section, comprising layers 1 through 5, the sediments are rich in benthos and clay and are thus interpreted as deposits of a tidal to shallow lagoonal environment, originating from the margins of the lagoon. The uppermost laminated part of the section (layers 7 and 8) is rich in carbonate and without any endobenthos. These uppermost layers represent the sediment deposits of a central part of the lagoon [18]. The pterosaur originates from the uppermost part of the section and was found in layer 6, partition 17.

**Table 1:** Ages of lithographic limestones in Southern Germany.

Stage	Zone	Subzone	Horizon	Localities
Tithonian	Palmatus		*palmatus*	
			*scoparius*	
	Ciliata		*callodiscus*	
			*ciliata*	
			*penicillatum*	Ellenbrunn
	Vimineus		*vimineus*	
	Mucronatum		*levicostatum*	
			*franconicum*	Gansheim, Störzelmühle
	Hybonotum	Moernsheimensis	*laisackerensis*	
			*moernsheimensis*	**Mörnsheim, Daiting**
		Rueppellianus	*rueppellianus*	**Solnhofen, Langenaltheim**
			*riedlingensis*	**Hienheim, Ried, Kelheim (Goldberg)**
		Riedense	unnamed	**Eichstätt (Schernfeld, Wintershof, Blumenberg)**
			*eigeltingense*	**Painten (upper part), Zandt**
Kimmeridgian	Beckeri	Ulmense	*rebouletianum*	Painten, Schamhaupten, Öchselberg, Walting
			*hoelderi*	Nusplingen
			*zio-wepferi β*	Nusplingen, Grosser Kirchbühl)
			*zio-wepferi α*	
		Setatum	*siliceous*	
			*uracensis*	Dollnstein (Torleite)
			*ornatum*	Beilngries
			*supinum*	Beilngries
			*minutum*	
		Subeumela	*fischeri*	
			*subsidens*	
			*kiderleni*	**BRUNN**
			*pedinopleura*	
	Pseudomutabilis	Pseudomutabilis	*semicostatum*	
			Not yet studied in detail	Wattendorf

Based on Schweigert (2007) Traditional Solnhofen localities are in bold, Brunn is in capitals.

## Methods

The speimen was described primarily under normal lighting regimes and examined with and without a light microscope and hand lens. Additional examination and photographs were then taken under UV lights by H.T.

**Figure 2:**
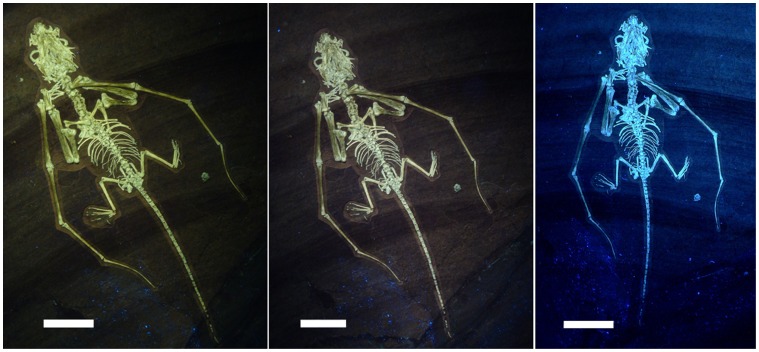
*Bellubrunnus* under multiple UV regimes. Different filter and light combinations illuminate the bones and matrix differently providing greater clarity of some details. A selection are shown here for reference. *Full page width.*

For a general introduction to the methods used here to visualise vertebrate fossils in ultraviolet-light (UV) light see [19]–[21]. For UV investigation of the specimen here we predominantly used UVA lamps with a wavelength of 365–366 nanometers. The use of a variety of different filters allows selective visualisation of some fine structures. A series of experiments led to the determination of the optimal filter combination, the displacement, intensity, and incident angle of the ultraviolet lamps (e.g. see Figure 2). Documentation via ultraviolet-light photography was executed by means of analogue photography on slide film as well as by digital photography.

No specific permits were required for the described field studies.

### Nomenclatural Acts

The electronic version of this document does not represent a published work according to the International Code of Zoological Nomenclature (ICZN), and hence the nomenclatural acts contained in the electronic version are not available under that Code from the electronic edition. Therefore, a separate edition of this document was produced by a method that assures numerous identical and durable copies, and those copies were simultaneously obtainable (from the publication date noted on the first page of this article) for the purpose of providing a public and permanent scientific record, in accordance with Article 8.1 of the Code. The separate print-only edition is available on request from PLoS by sending a request to PLoS ONE, 1160 Battery Street, Suite 100, San Francisco, CA 94111, USA along with a check for $10 (to cover printing and postage) payable to “Public Library of Science”. The online version of the work is archived and available from the following digital repositories: PubMedCentral, LOCKSS.

In addition, this published work and the nomenclatural acts it contains have been registered in ZooBank in the proposed online registration system for the ICZN. The ZooBank LSIDs (Life Science Identifiers) can be resolved and the associated information viewed through any standard web browser by appending the LSID to the prefix “http://zoobank.org/”. The LSID for this publication is: urn:lsid:zoobank.org:pub:7D0C6675-1B0B-4A22-8545-7E56BEE93909.

### Description

**Figure 3:**
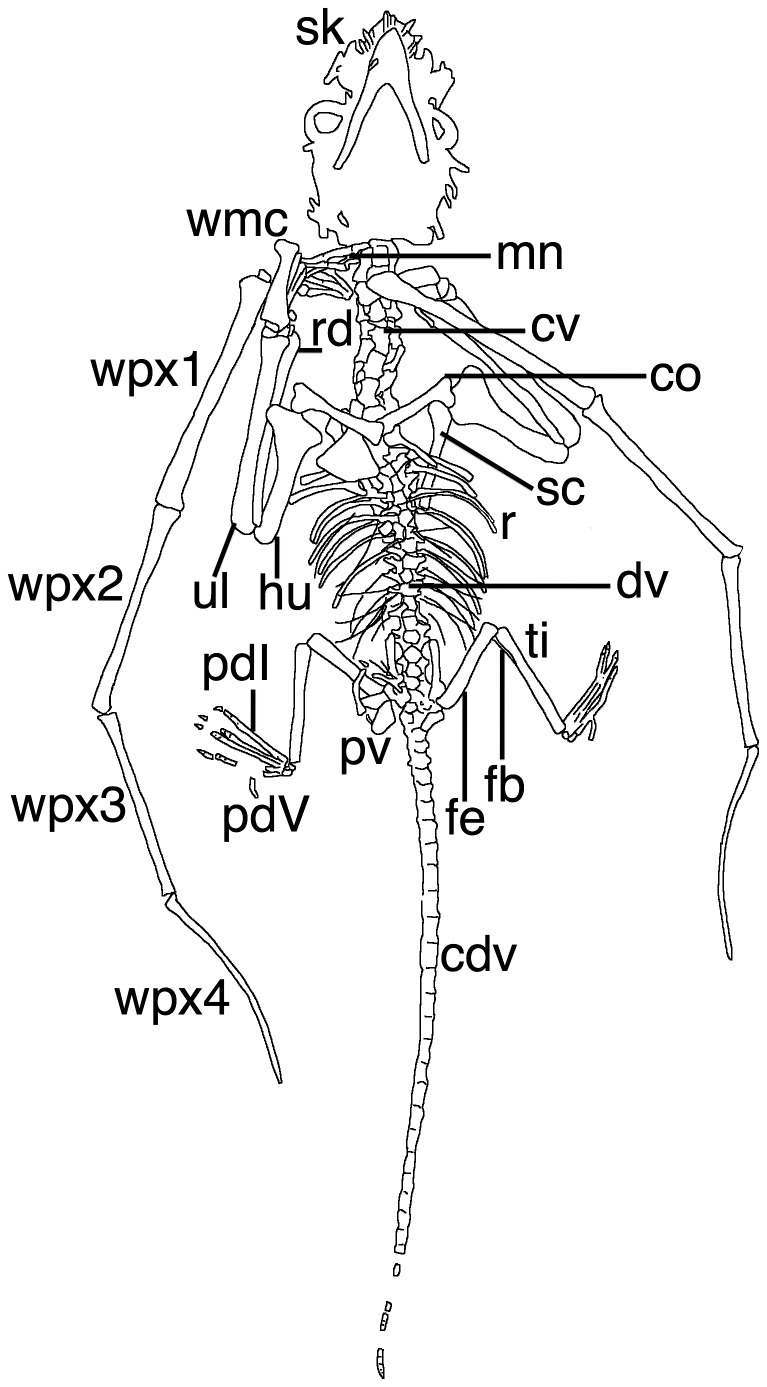
Line drawing of the holotype of *Bellubrunnus*. Abbreviations as follows for this and, where appropriate, subsequent figures: cdv, caudal vertebrae; chv, chevron; co, coracoid; cp, carpus; cs, cristospine; cr, cervical rib; cv, cervical vertebrae; dr, dorsal rib; dv, dorsal vertebrae; fb, fibula; fe, femur; g, gastralium; hu, humerus; il, ilium; ish, ischium; mc, metacarpal; md, manual digit; mn, manus; pb, pubis; pd, pedal digit; ppb, prepubis; ptd, pteroid; r, ribs; rad, radius; sc, scapula; sk, skull; st, sternum; ul, ulna; wmc, wing metacarpal; wpx, wing phalanx. *Single column width.*

The specimen is a complete and articulated skeleton of a juvenile non-pterodactyloid pterosaur seen in ventral view (see Figures 1, 2, 3). The specimen is very well preserved and the details of even the smallest bones such as the palatal elements, tarsals and gastralia are clear, despite the young age and small size of the specimen (see Table 2 for measurements). There is no evidence of any soft-tissue preservation on the specimen, even after investigation under UV light (see Figure 2). Figure 2 clearly shows that some elements are absent from the right pes and distal tail, though external moulds of the bones in the matrix show that these were originally present. Some parts of the specimen have disarticulated slightly (e.g. the cranium, some middle dorsals) and the skull is crushed dorsoventrally to a degree. Overlapping elements are, in places, difficult to distinguish from one another or cannot be identified accurately.

**Table 2:** Key measurements of the holotype of *Bellubrunnus* to the nearest mm.

Element/Anatomical section	Length (L/R) mm
Skull	23 (13 wide at base of skull)
Cervical series	19 (last cervical obscured)
Dorsal series	25
Sacral series	4
Caudal series	71
Sclerotic ring (left)	External diameter 6, internal diameter 3
Coracoid	9/10
Scapula	11/12
Humerus	14/14
Ulna	22/22
Metacarpal IV	9/9
Phalanx IV-I	27/27
Phalanx IV-II	23/23
Phalanx IV-III	20/21
Phalanx IV-IV	23/23 (measured in straight line)
Femur	10/10
Tibia	12/12
Metatarsal I	6/6
Sternum	8 (7 wide)

**Figure 4:**
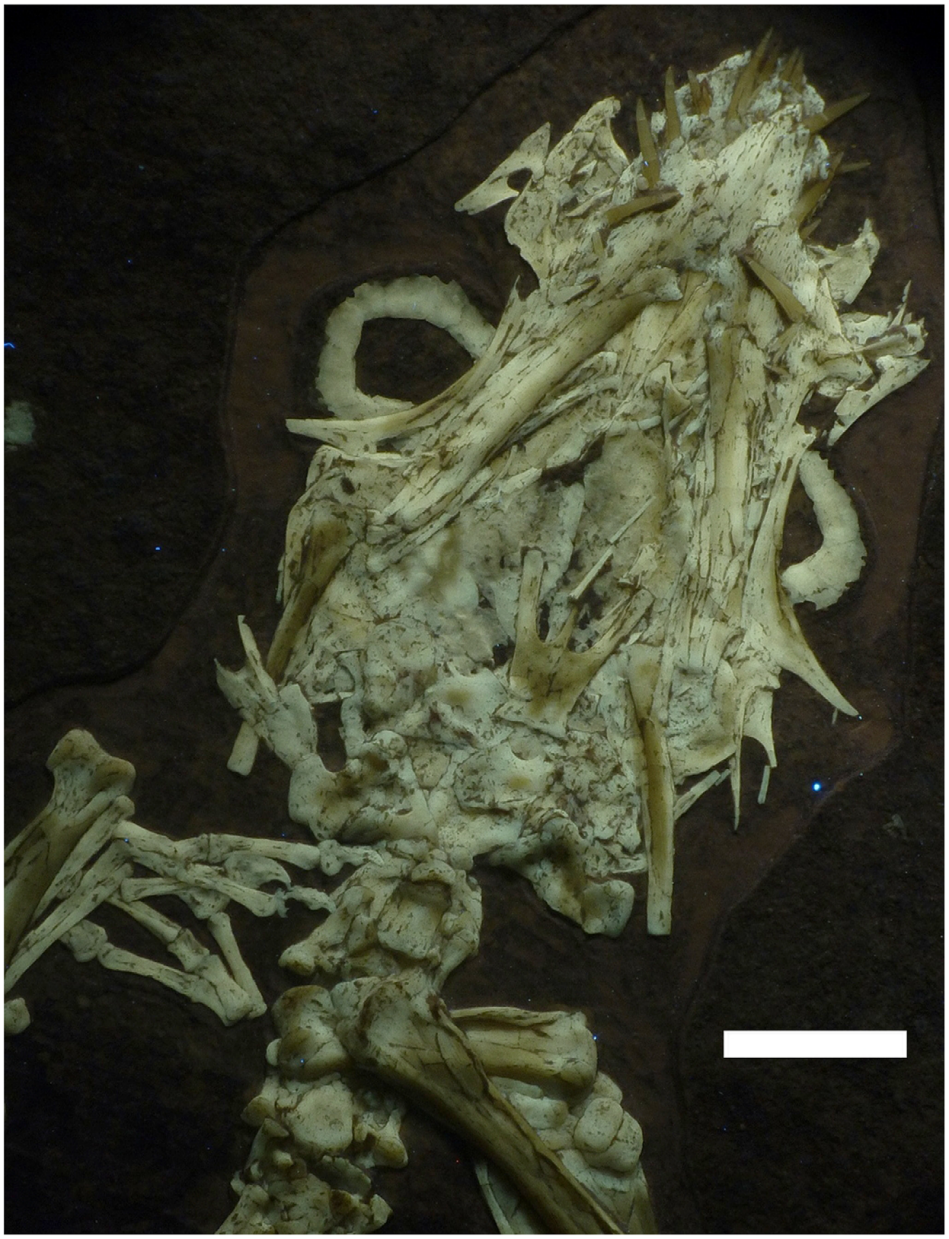
Close up of the skull of *Bellubrunnus* under UV light. Scale bar is 5 mm. *Column and a half width.*

**Figure 5:**
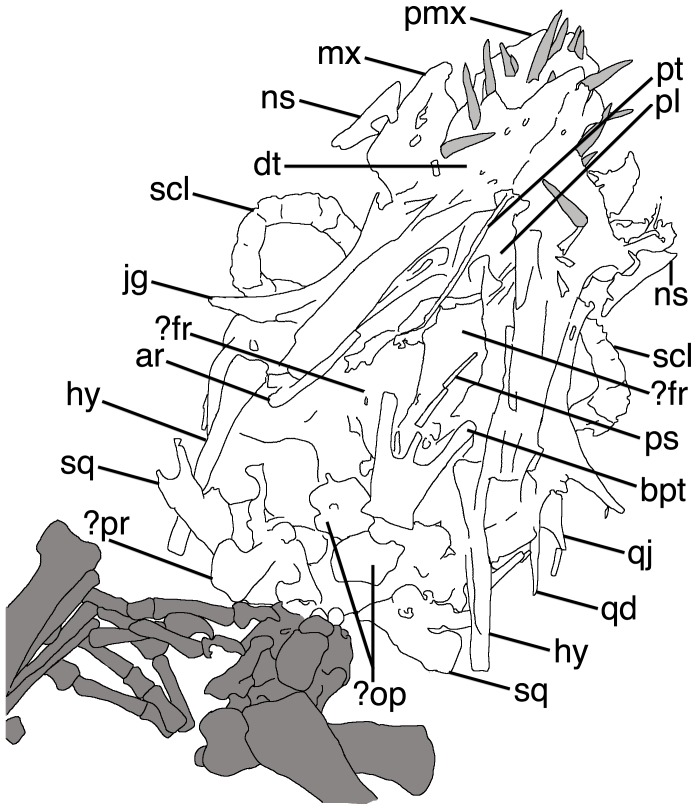
Line drawing of the skull of *Bellubrunnus.* The teeth are shaded pale grey and the non-cranial elements are in dark grey. Abbreviations as follows: ar, articular; bpt, basipterygoid; dt, dentary; fr, frontal; hy, hyoid; jg, jugal; mx, maxilla; ns, nasal; op, opsithotic; pl, palatine; pmx, premaxilla; pr, prootic; ps, parasphenoid; pt, pteryoid; qd, quadrate; qj, quadratojugal; scl, sclerotic ring; sq, squamosal. *Single column width.*

The skull is especially difficult to interpret. As seen in ventral view, the mandible overlaps the palate that in turn lies over the skull roof. To add further complication, some elements have disarticulated and moved a little from their natural positions. Some elements may have been broken or distorted under compaction, and some sutures between elements are incomplete or unclear, probably due to incomplete ossification. Some of the identifications of skull and mandibular elements must therefore be regarded as tentative (Figures 4, 5).

The skull is triangular in outline in ventral view with a gentle tapering to a rounded anterior terminus (Figures 4, 5). Despite being somewhat disarticulated, it appears to be complete and with all elements preserved. The premaxillae have a rounded anterior margin, however owing to the position of the teeth, the interpremaxillary suture and premaxilla-maxillary sutures cannot be seen. The maxillae have turned out from the skull so each is seen in lateral, and not ventral, view. The flat triangular maxillae extend posteriorly to at least level with the rostral margin of the orbit. As with the maxillae, the small, sub-triangular nasals lie off to the sides of the skull. The nasals are small sub-triangular elements, but have suffered some damage as their form is difficult to make out.

Both jugals are seen in lateral view, though their ventral margins are obscured by the dentaries (Figure 5). The jugals are boomerang-shaped elements, each with a long posterodorsally directed process that forms the posterior margin of the orbit. The dorsal margins of the jugals form the ventral margin of the orbits. The orbits themselves are large and presumably sub-circular in outline based on the visible shapes of the elements that would make up this opening (Figure 4). A small quadratojugal is preserved posterior to the jugal on the left, the right quadratojugal may also be seen, though this identification is tentative (Figure 5). The quadratojugal is sub-triangular with a broad base tapering to a thin dorsal ascending process. Both quadrates are preserved in ventral view and again the left is more clearly visible. Each has a large expansion medially directed ala that forms the posterior part of the palate, with a long, thin and tapering dorsal ascending process that lies alongside the quadratojugal. Both squamosals are seen in ventral view with the right being better preserved. The right element is rather plate-like with a forked dorsally projecting process. A piece of bone lying posterior to the right squamosal may represent the right parietal (Figure 5).

A large part of the skull roof can be seen to be *in situ* below the mandible and palate. Individual bones cannot be identified, but based on other rhamphorhynchids, this is presumably mostly formed by the dorsal rami of the premaxillae and the frontals (Figure 5).

The palate is largely intact though elements have broken and moved (Figure 4). The difficulty of identifying individual bones, especially in the posterior region, is compounded by the apparent disarticulation of the braincase and the fact that the palate lies on top of the skull roof. Thus more elements than identified here are probably present, but we are unable to diagnose them. Anteriorly a pair of large and plate-like palatal bones lie together and form the roof of the mouth posterior to the premaxillae. These palatals have a concave posterior margin and together form the U-shape cranial margin of the palatine foramen the level of the midpoint of the orbits. Some forked and splint-like pieces of bone lying across the posterior parts of the palatines most likely represent the disarticulated pterygoids, though their original form cannot be determined (Figure 4, 5).

The parasphenoid lies in the median line of the palate posterior to the orbits. It is a long, thin element that strongly tapers anteriorly to a point. The paraspenoid is broken in several places but otherwise is complete (Figure 4). Posteriolateral to the paraspenoid there is a pair of basipterygoids that also taper anteriorly. Together with the median rostral process of the paraspenoid the basipterygoids form a trident-like structure, with the lateral rami diverging at an angle of approximately 20° from the midline (Figure 5). Posterior to the basipterygoids lie the paired opisthotics that have separated from the braincase and lie on top of the basisphenoids and where the foramen magnum should be. The opisthotics are tentatively identified based on their position, though in adult rhamphorhychines these are of a similar shape to the prootics [8] and the two may be confused here (Figure 5). The elements identified as opisthotics are seen in posterior view, though given their respective positions, the left element seems to have rotated 180° around its middle, and the right is damaged being pierced in the central part of the bone. Each opisthotic consists of a vertical semi-circular corpus with a convex lateral margin. From the anteriolateral margin of the corpus a straight anteriolaterally directed process arises.

The mandible is preserved in articulation with the skull and the contralateral rami of the dentaries are fused anteriorly in the midline to form the symphysis (Figure 4). The tip of the lower jaw is rather pointed at a near right angle and teeth are present close to the anterior tip of the mandible. The symphysis of the two dentaries runs for the entire length of their association. Posterior and ventrally to the dentaries are two rami of bone considered to be the articulars. These are much shorter than the dentaries and rectangular in profile. Overall the mandible appears to be rather short compared to the length of the skull (Figure 4). However, the mandibular articulation point of the jaw with the skull is estimated to have lain ventral to the middle of the orbit. Thus the mandible is not unusually short, but the skull appears long owing to the flattening out of the posterior face of the skull. Given the condition of the skull it is most likely that all elements of the mandible are present however, only a putative articular from the right side can be identified with any confidence.

A total of 21 teeth are visible in the skull and mandible, and thus presumably the animal had 22, assuming all teeth were paired. It is not clear however, which teeth belong to which tooth bearing skull element (of the premaxillae, maxillae and dentaries). This is likely to represent most of the original teeth as in rhamphorhynchid pterosaurs the teeth are partly directed out from the jaws, such that during dorsoventral compression of the cranium the teeth should be spread out around the jawline and be visible. Even if the teeth turned inwards, these should then be visible on top of the palate. The largest teeth are those of the anterior part of maxilla and the anterior dentary. Teeth are smaller in the premaxilla (about half the length and width of the largest teeth), and only slightly smaller that these in the posterior part of maxilla and dentary. One tooth in the prexmailla appears to underlie that at the very tip and may represent an incipient replacement tooth. If so, then the true tooth count would be 20. All teeth are simple, spike-like and appear to be sub-circular in cross-section (Figure 4). The teeth are either straight or only slightly curved, and none are fang-like or heavily curved as in a number of other non-pterodactyloids (e.g. *Angustinaripterus, Dimorphodon, Rhamphorhynchus*).

Both sclerotic rings are present (Figure 4). They appear to be fused into single units, but close examination shows that the individual sclerites are indeed separate and there would have been about 14 in each eye. Based on the size of the sclerotic rings and the shape of the jugals, the eyes would have been large in comparison to the size of the skull, being nearly as tall as the skull and about one third of the length. They also suggest that the skull would have been tall compared to its total length (close to 3∶1) as is seen in other juvenile specimens of rhamphorhynchines. Two hyoid bones lie posterior to the mandibular rami. Each is long and straight and rather robust, being around one tenth as long as wide (Figure 5).

**Figure 6:**
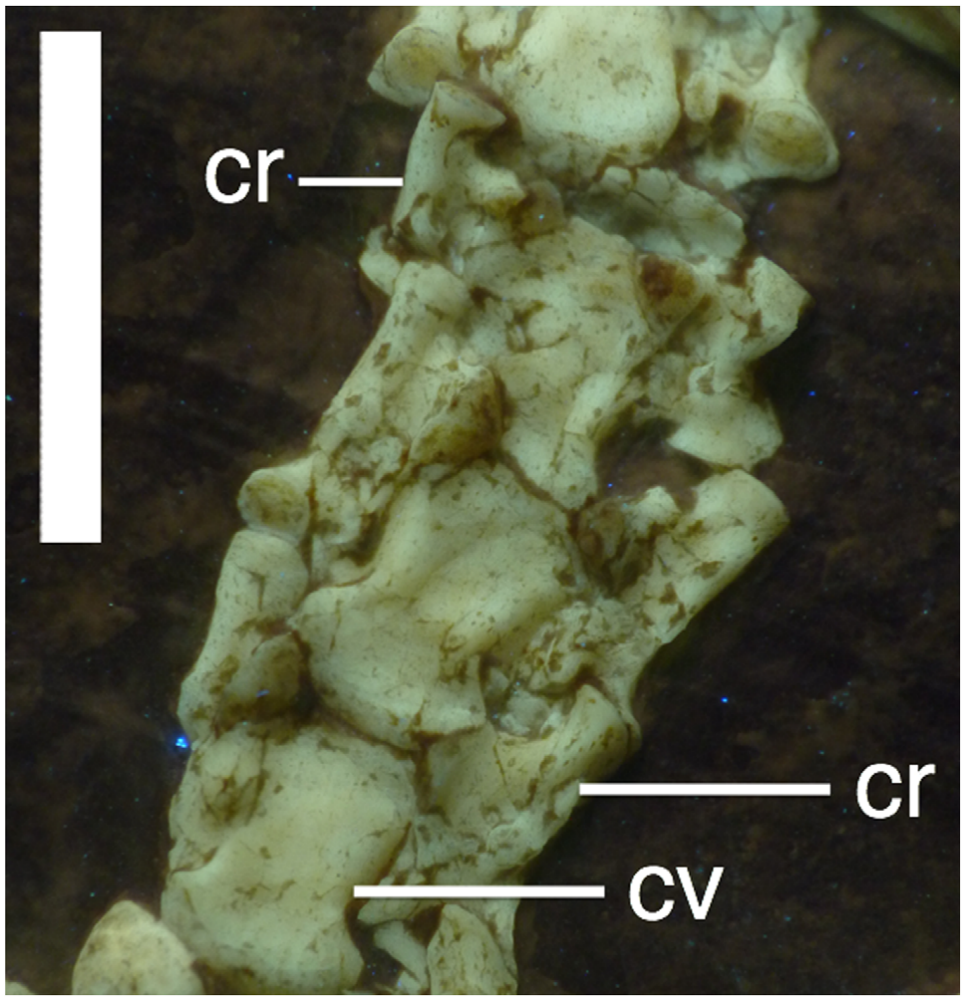
Cervical vertebrae of *Bellubrunnus* (anterior to the top) under UV light. The unfused cervical ribs are clearly visible to each side of the column. Scale bar is 5mm. *Single column width.*

At least seven cervical vertebrae are present, (not including the atlas/axis complex which is not visible and is most likely hidden by the back of the skull) (Figures 4, 5, 6). They are slightly longer than broad and the centra appear to be cylindrical with slightly expanded ends (Figure 6). The posterior cervicals have a slight constriction in the posterior part of the centra. The cervical ribs are not fused to their respective centra at the anterior end of the lateral surfaces. The cervical ribs are short (less than the length of the respective centra) and are robust with flat anterior and posterior ends (Figure 6).

**Figure 7:**
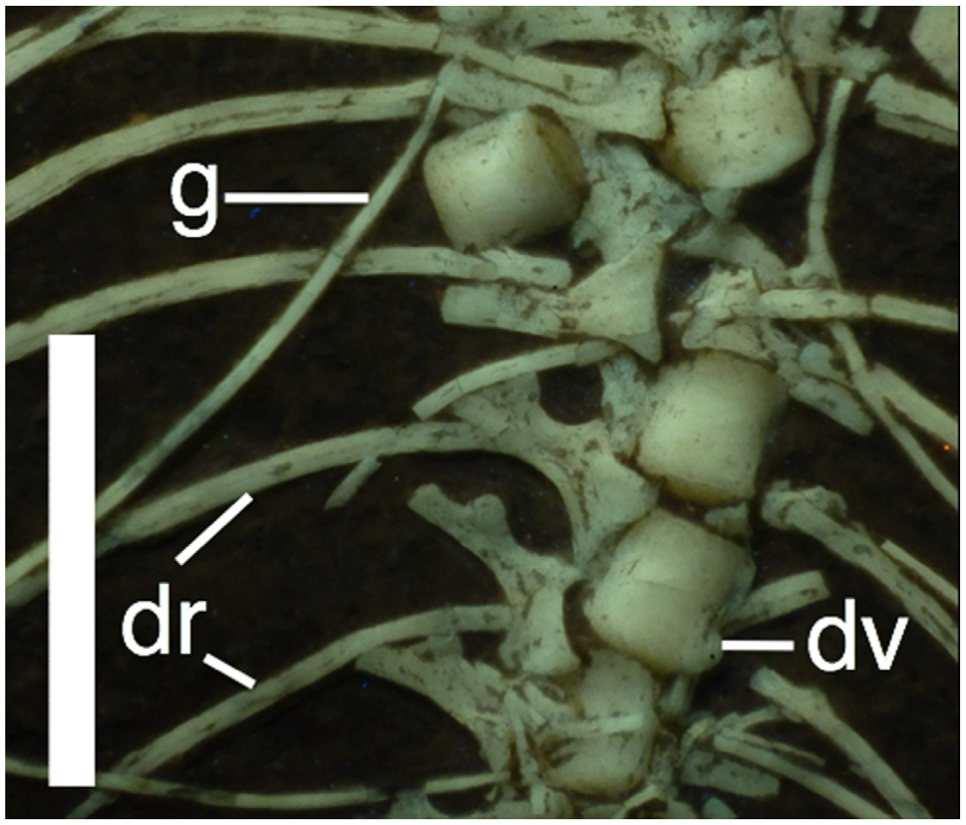
Dorsal vertebrae and ribs of *Bellubrunnus* (anterior to the top) under UV light. Note the displaced single centrum and the lack of fusion between centra and neural arches. Scale bar is 5 mm. *Full page width.*

**Figure 8:**
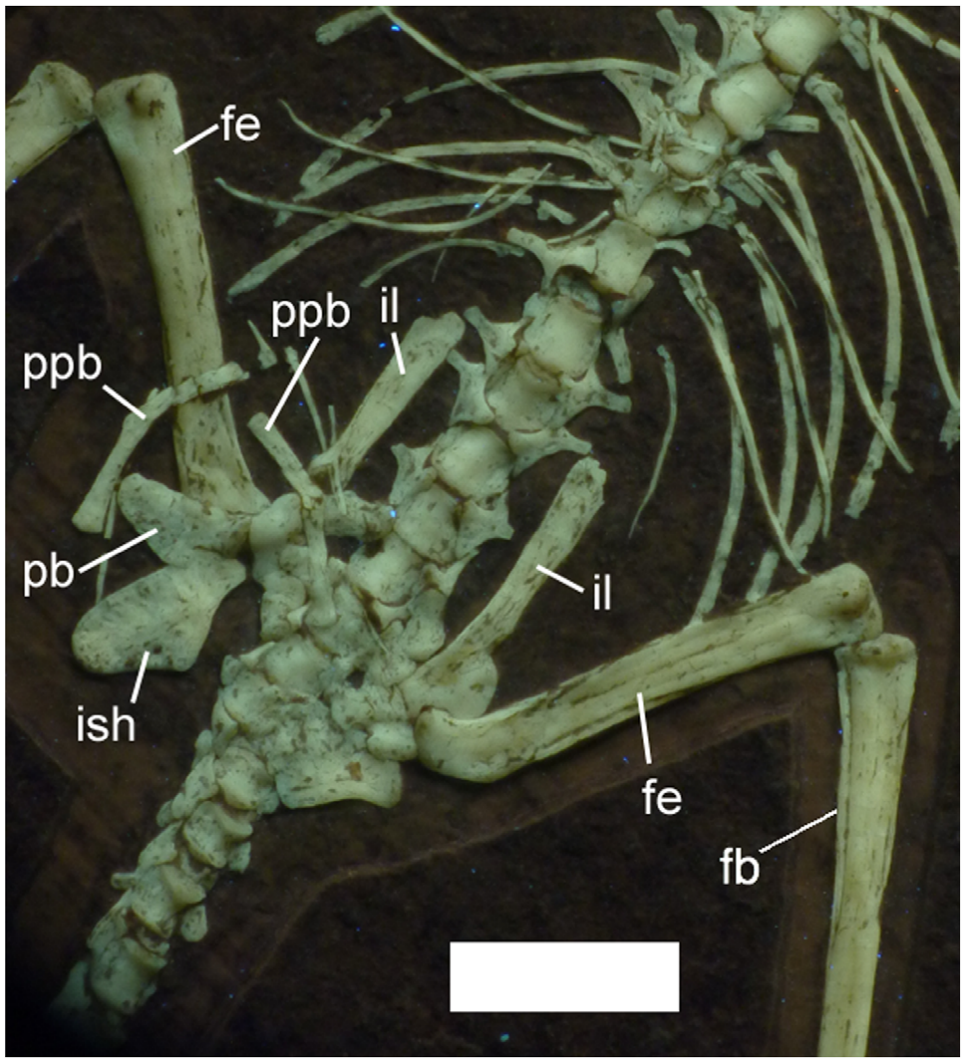
Pelvis of *Bellubrunnus* under UV light. Note the disarticulation of the major elements. Thin splint-like elements lying anterior to the prepubes are probably gastralia. Scale bar is 5 mm. *Single column width.*

There are approximately 17 dorsal vertebrae present. The transition from cervicals to dorsals is hidden by the coracoids making the exact number of each difficult to determine. Most of the dorsals are in their natural articulation, though some of the mid dorsal centra have separated from each other and moved a short distance laterally (Figure 7). In each case the neural arch remains in place, demonstrating that the centra were not fused to their respective neural arches. The first 13 dorsals on each side are associated with dorsal ribs. The dorsal ribs are dicephalic and curved and gently taper to a point distally (Figure 7). The distalmost dorsals are bounded by the anterior wings of the ilia (Figure 8) and do not have ribs associated with them.

**Figure 9:**
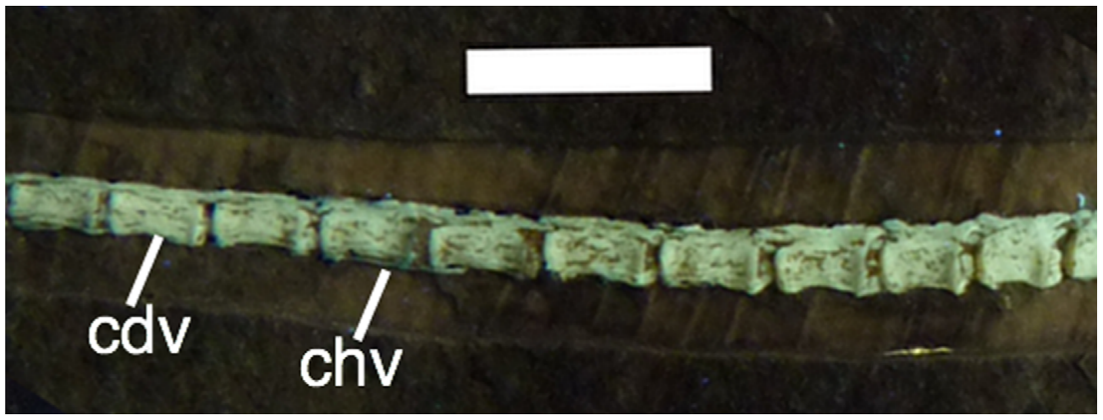
Caudal vertebrae of *Bellubrunnus* (anterior to the right) under UV light. Scale bar is 1 cm. *Full page width.*

At least five gastralia are present along the middle to posterior part of the dorsal vertebral series (Figure 9). These gastralia are not fused in the midline and indeed most have displaced to the right side of the specimen and lie perpendicular to the dorsal column. The position of these lying close to the dorsal ribs and well lateral to the midline of the dorsal column suggests that these elements may be sternal ribs. However, we reject this interpretation as, despite their position, their morphology is entirely consistent with gastralia and quite unlike the short, broad, and serrated outline of pterosaur sternal ribs (e.g. [22]).

Three sacrals are inferred to be present (Figure 8). Although these are not fused to each other, or the ilia, these are considered as sacrals because they have broader centra than the dorsals. These also possess broad lateral processes, and are therefore sacral ribs.

Thirty-eight caudals are present (including the terminal caudal which is distinguished by having a rounded posterior tip). One distal caudal is missing from the slab but a mould on the matrix shows that it was previously present. The caudals are seen in right lateral view and the neural arches appear to be dorsoventrally very short and not fused to the centra (Figure 9). Only short (each less than half the length of the centrum), rapidly tapering pre- and postzygapophyses are present. Only a small number of chevrons are present. The chevrons are long, thin and splint-like elements and are not bound to the vertebrae or interleaved with each other as in many basal pterosaurs (Figure 9). Those preserved are only two to three times the length of the caudal centra, unlike those seen in *Rhamphorhynchus* which are more than five times longer [8].

**Figure 10:**
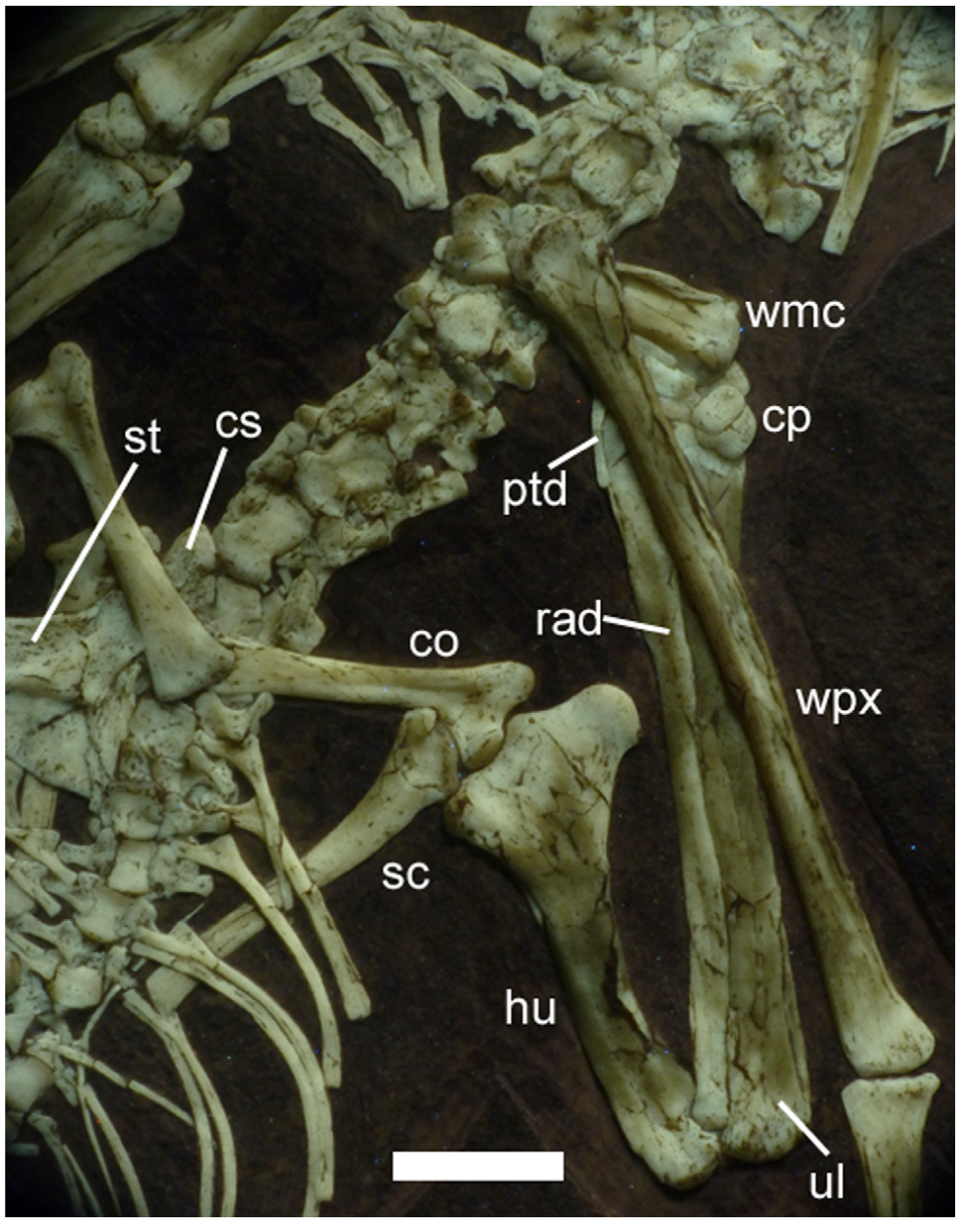
Shoulder girdle, proximal forelimb and sternum of *Bellubrunnus* under UV light. Scale bar is 1 cm. *Single column width.*

The scapulae and coracoids are not fused into their respective scapulocoracoid pairs and lie slightly apart from each other in each case (Figures 1, 10). As the right sided elements are overlapped by the sternum, the description here is based on the left scapula and right coracoid. Both the scapula and coracoid are long and straight with slightly expanded proximal ends, although this expansion is more pronounced in the coracoid, and the scapula is the slightly longer element (Figure 10). Both bones are enlarged distally to form the genoid fossa where the elements meet, which is again larger in the coracoid. There is also a slight and rounded ?laterally facing projection from the distal end of the coracoid as also seen in *Rhamphorhynchus* [8].

The sternum has a large cristospine that extends anteriorly and is nearly as long as the body of the sternum itself (Figure 10). The elongate cristospine is seen in at least one other small specimen of *Rhamphorhynchus* – CM 11433 (CM: Carnegie Museum of Natural History, Pittsburgh, U.S.A.). The overall shape in *Bellubrunnus* is also similar to the condition in *Scaphognathus* [23] but lacks the large paired foramen of this and other related taxa (e.g. [24]). The sternal plate is fan-shaped with a convex posterior edge. The sternum is relatively small element and is proportionally smaller than that seen in some basal pterosaurs [23].

The humerus is straight, with no curvature to the distal part of the shaft that is seen in many non-pterodactyloid pterosaurs including small *Rhamphorhynchus* specimens [8] preserved in a similar orientation. The deltopectoral crest is large (being slightly wider than the humeral shaft) and projects a long way from the shaft (Figure 10). This has a semicircular profile and ventrally it tapers gently into the midshaft of the humerus. The medial crest of the humerus is about half the size of the deltopectoral crest and is subtriangular in profile, though the distal tip is well rounded. The proximal and distal ends of the humerus are flat as also seen in *Rhamphorhynchus*, though there is a slight rounded projection on the proximal face.

The radius and ulna are subequal in length and appear to be similarly robust (Figure 1). These are simple elements and are long and straight, with slightly enlarged proximal and distal ends.

The elements of the carpus are slightly disarticulated (more so on the left arm) and are not fused together as in adult pterosaurs, all elements appear to be present however (Figure 10). A number of carpals are present in each wrist but are slightly disarticulated and are somewhat amorphous in shape making exact identities difficult to determine. The separation, small size and large number of carpals present (at least four elements are visible in the right wrist, in addition to the pteroid) suggest that these have yet to fuse into the proximal and distal syncarpal pair normal for adult pterosaurs. The carpals present vary in size but all are relatively small and are somewhat amorphous in shape, having yet to fully ossify. Both pteroids are preserved but are partially hidden by other elements (Figure 10). They are short, thin and straight rods of bone.

**Figure 11:**
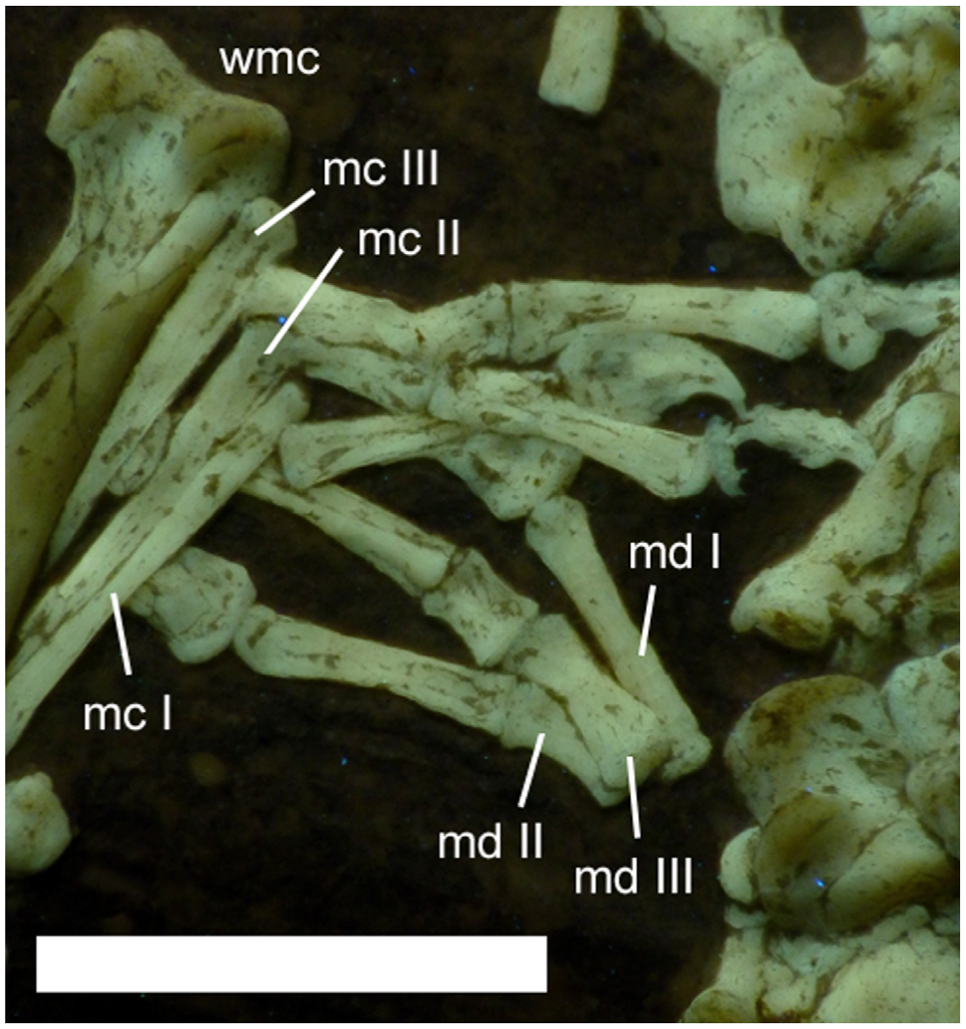
Hands of *Bellubrunnus* under UV light with the right overlying the left. Individual phalanges are not labelled for clarity. Note the damage to the ventral part of the ungual of digit 3 on the right hand. Scale bar is 5 mm. *Single column width.*

The metacarpals of digits I-III are slightly separated from each other and from the large wing metacarpal (IV) (Figure 11). The phalanges of digits I-III of the two hands overlap each other and are also somewhat disarticulated, making it difficult to determine individual elements exactly. The phalangeal formula of the hand is 2-3-4-4-X. The manual phalanges of digits I-III are thin, straight elements. The manual unguals of digits I-III are short and robust (dorsoventrally about twice the height of the phalanx with which they articulate), and have only a slight curvature.

**Figure 12:**
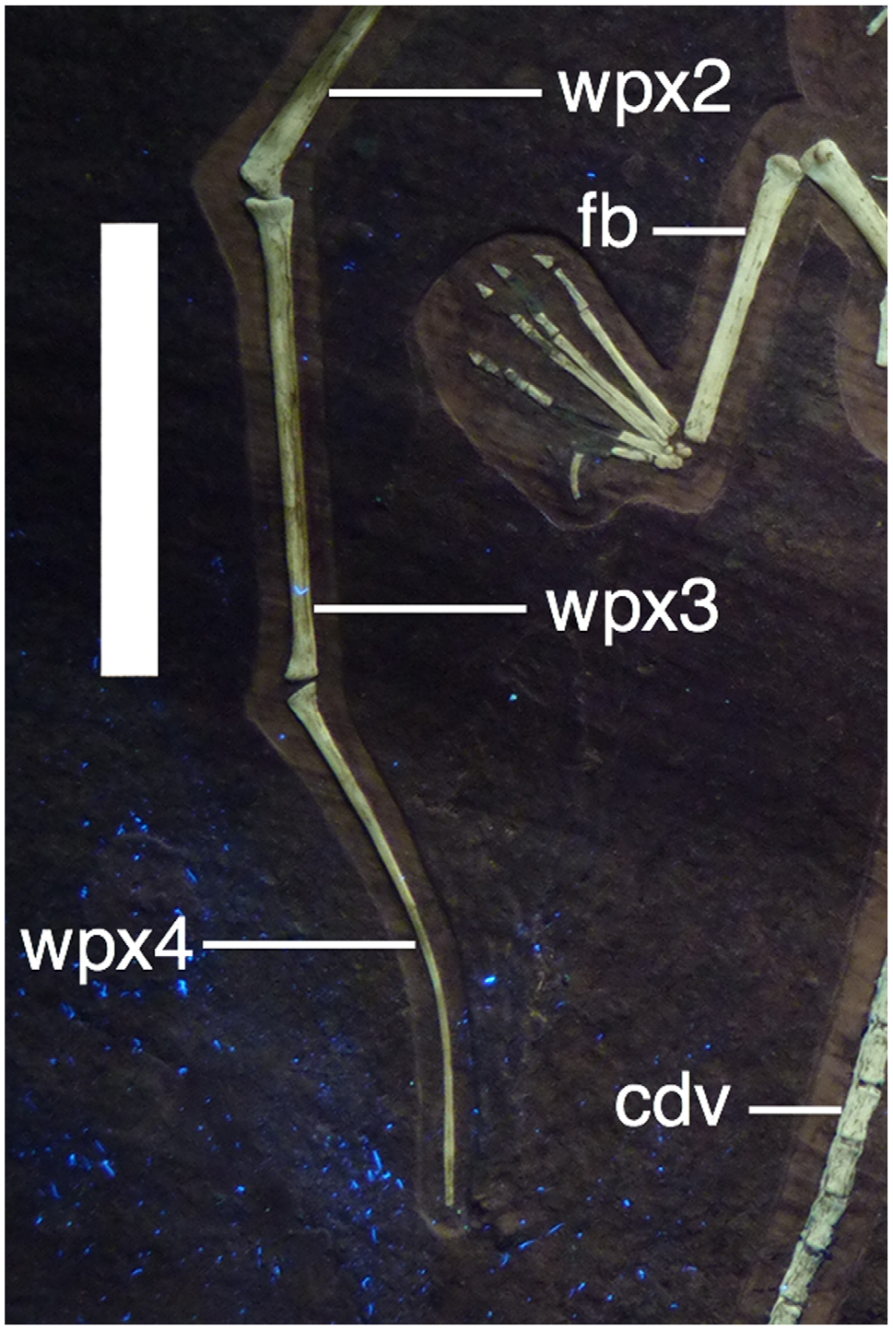
Right distal wing of of *Bellubrunnus* under UV light showing the pronounced curvature of the fourth wing phalanx. Also visible is the fact that wing phalanx 3 has rotated along its long axis. Scale bar is 2 cm. *Single column width.*

Metacarpal IV is robust and tapers strongly towards its distal end before the enlarged ginglymoid distal end (Figures 10, 11). The extensor tendon process is not fused to wing phalanx 1 and has moved slightly from its normal position. The right lies at the proximal end of wing phalanx 1 and can be seen to be sub-oval in shape. The wing finger phalanges are generally straight, except for the fourth on both wings which is gently curved anteriorly along their length and terminates in a point (Figures 1, 12). Unusually wing phalanx 4 is longer than 3, such that the lengths of the phalanges are 1>2 = 4>3. The bones of each wing finger have rotated along their long axes, though they have not disarticulated. This is quite clearly seen as the posterior face of the ends of pterosaur wing finger bones are expanded whereas the anterior is not. Thus it can be seen that in the left wing, phalanx 4 is rotated 180° along its long axis relative to the others, and in the right wing, phalanx 3 has undergone the same rotation (Figure 1).

All the elements of the pelvis are present (Figure 8). The right pubis, prepubis and ischium are more clearly visible than the left and the description is based on these elements. The ilium, ischium and pubis are not to each other fused and the iliosacral suture is open (Figure 8). The ilium has a long anterior process that is slightly expanded anteriorly and has a rounded anterior end. The prepubis is a thin bifurcated element. Together with the contralateral element, the prepubic bones would meet form an almost ‘H’ shaped structure. The pubis is plate-like with a straight anterior edge, a posteriordorsally angled ventral edge and then a slight anterior invagination on the posterior face, just ventral to the acetabulum (Figure 8). The anterior face is flat and slightly expanded laterally. The ilium has an outline similar of to the pubis but in a mirror-image and is slightly larger. The pubis and ischium should meet such that their ventral two thirds would not touch leaving a deep ‘V’ in the ventral part of the lateral face of the pelvis.

The femur is short relative to the humerus (see below). The head is offset medially from the shaft at about 90° and there is no constricted neck at the base of the femoral head (Figure 8). The shaft is straight and the distal end is only slightly expanded relative to the shaft. The tibia is a simple and straight element, which tapers slightly (about ¾ of the proximal diameter) from a broad proximal end. The proximal part of the left fibula is partly visible as a thin splint of bone running alongside the tibia (Figure 8).

**Figure 13:**
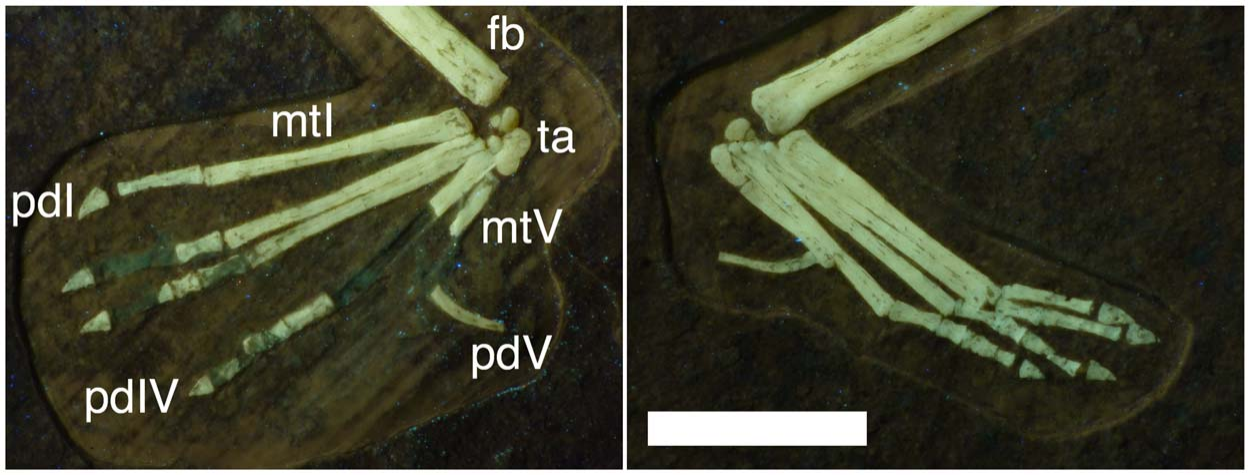
Composite of the feet of *Bellubrunnus* under UV light as on the slab (such that the right foot lies on the left of the photo and vice versa). Note that on the right foot some bones are missing but impressions of them remain. The ankle elements are preserved despite their extremely small size. Scale bar is 5 mm. *Full page width.*

At least two tarsals are present on each pes (two are visible on the left foot and three on the right) (Figure 13). They are not fused together or to the tibia. The largest most likely represents the astragalus given their size and position, but all are rather amorphous since they have yet to fully ossify. The others are much smaller and likely represent the calcaneum and on the right foot, a single distal tarsal. Distal tarsals are often un-ossified even in large and near adult pterosaurs and it is notable that they are preserved here.

Both pedes are near complete (Figure 13). Only a few elements of the right pes are missing, but are identified by moulds in the matrix. Much of the left pes appears to be missing but is clearly visible with illumination under UV (see Figures 1, 2, 13). Metatarsals I-IV are long and straight with I and II being slightly longer than III and IV being a little further reduced. Metatarsal V is much shorter than I-IV and is slightly curved. The pedal formula is 2-3-4-5-2 as is typical for non-pterodactyloid pterosaurs. The phalanges are unremarkable, being simple, straight elements expect those of digit V, which lacks an ungual and has a characteristic ‘boomerang’ shaped second phalanx as do other rhamphorhynchids, though the curvature is far less than many other species. The unguals on digits I-IV are short and only slightly curved and are not dorsoventrally deep as in the manual unguals.

## Discussion

### Ontogenetic Status

In addition to body size, Bennett [9] identified a number of characteristics that marked out specimens of *Rhamphorhynchus* as juveniles, many of which are present here. As a close relative (see below), these juvenile characters are thus appropriate as diagnostic indicators of a young age of the *Bellubrunnus* holotype.

The orbits are very large and the skull is proportionally short and broad, as are the edentulous jaw tips with a blunt tip to the lower jaw. The texture of the bones is rough-porous and grainy-granulated seen in other juvenile pterosaurs [9]. Under UV light (see Figures 2, 12), some of the shafts of the long bones, notably the humeri, ulnae, and the wing finger phalanges 1 and 2 also fluoresce rather less that other skeletal elements suggesting that these were less ossified than other elements. However, no epiphyses are present on the ends of long bones as is often seen in non-adult pterosaurs, (e.g. *Anhanguera* – [25]) and the sternum is present and fully ossified as indeed are the small tarsals. As with the juveniles of most tetrapods, many bones are not fused together. The bones of the skull and palate are unfused, the carpals and tarsals are unfused, and the respective pairs of scapulae and coracoids are separate. According to Bennett [9] the fusion of the scapulocoracoid occurs around the end of the first year in *Rhamphorhynchus* suggesting that this is an animal less than one year old. The cervical ribs are not fused to their respective vertebrae, and the neurocentral sutures are not closed in the vertebral column as a whole (e.g. Figure 7). The elements of the pelvis are separate from one another and the sacral vertebrae are neither fused to one another, nor to the ilium.

Measurements of the skeleton (See Table 2) put the holotype of *Bellubrunnus* as among the very smallest pterosaur specimens known, and in size also corresponds to Bennett’s [9] year class 1 of *Rhamphorhynchus*. *Bellubrunnus* is smaller in wingspan than the young *Anurognathus* specimen described by Bennett [5], *Changchengopterus* [26] and similar to, or smaller than, *Eudimorphodon ranzii* [27] and the tiny pterodactyloid *Nemicolopterus* [28]. This was then, a very young individual.

### Taxonomic Assignment

*Bellubrunnus* is identified as a rhamphorhynchid pterosaur [29] as there are less than 11 pairs of teeth in the rostrum and a tongue shaped deltopectoral crest of the humerus with a constricted base (though here the base is reduced). The specimen is further defined as a member of the Rhamphorhynchinae based on the following characters [29]: mandible tips fused into a short symphysis bearing a forward-projecting prow and a number of large, fang-like, procumbent teeth; wing finger 63% or more of total wing length (here it is over 67%). Two further diagnostic characters, from Unwin’s [29] definition (the shape of the antorbital fenestra and the groove on the posterior face of the wing finger) cannot be seen here. The inferred systematic position for *Bellubrunnus* is further supported by the following characters, erected by Kellner [30] for the Rhamphorhynchidae (which is near synonymous with Unwin’s [29] Rhamphorhynchinae): deltopectoral crest of humerus expanded distally in hatchet-shaped form and positioned near caput humeri and the last phalanx of pedal digit V elongated and curved.

Comparisons to rhamphorhynchine taxa *sensu* [2], [29] also show that *Bellubrunnus* is a distinct taxon. The proportions of major elements and series of elements of *Bellubrunnus* are similar to *Rhamphorhynchus*, but significantly different from those of other all other rhamphorhynchines (see Table 3). The reduced tooth count (20–22 teeth in total) serves to separate *Bellubrunnus* from *Rhamphorhynchus* (see below). The lack of the elongate chevrons and zygopophyses in the tail separates *Bellubrunnus* it from *Nesodactylus* [31], *Dorygnathus* and *Rhamphorhynchus*. It can be further separated from the recently revised diagnosis of *Dorygnathus* [32] by having second and third wing finger phalanges of different lengths and an ulna shorter than wing phalanges 2 and 3, and a wing phalanx 4 longer than the third. *Bellubrunnus* can be further separated from *Nesodactylus* by having a scapula longer than the coracoid [31] and from *Angustinaripterus* ZDM T 8001 (ZDM: Zigong Dinosaur Museum, Zigong, China) by lacking the massively elongate and recurved teeth in the anterior premaxilla of this Chinese taxon.

**Table 3:** Proportions of the lengths of major elements and series of *Bellubrunnus* and various rhamphorhynchines.

Taxon	Humeral length mm	Forelimb/Hindlimb	Humerus/Femur	Ulna/Tibia	Humerus/Metacarpal IV ratio
Rhamphorhynchinae	-	3.40–5.49	1.03–1.48	1.25–1.81	0.39–0.68
*Rhamphorhynchus* (1) TM 6924	16.5	6.55	1.32	1.72	0.60
*Rhamphorhynchus* (1) BSP 1889 XI.1	15.5	6.15	1.32	1.72	0.61
*Rhamphorhynchus* (1) MB	14.5	6.17	1.32	1.60	0.54
*Rhamphorhynchus* (1) MMK V45/1	13.5	6.14	1.23	1.62	0.63
*Rhamphorhynchus* (1) UB E 554	21.8	6.32	1.28	1.70	0.63
*Rhamphorhynchus* (1)	19	6.89	1.19	1.86	0.53
*Rhamphorhynchus* (2) MLU	33	6.91	1.18	1.56	0.58
*Rhamphorhynchus* (2) SMD	34	6.72	1.21	1.45	0.53
*Dorygnathus* BSP 1938 149	51	4.18	1.21	1.38	0.49
*Dorygnathus* UUPM R 157	61	4.34	1.22	1.48	0.48
*Quinglongopterus* D3080	17.8	6.07	1.46	1.85	0.93
*Nesodactylus* AMNH 2000	46.5	–	–	–	0.57
***Bellubrunnus***	**14**	**6.27**	**1.40**	**1.83**	**0.64**

Data for the clade Rhamphorhynchinae taken from Unwin, 2003; various specimens of *Rhamphorhynchus* from Wellnhofer, 1975 (numbers in parentheses represent Bennett’s [1995] size classes); *Dorygnathus* from Padian, 2008; *Qinglongopterus* from Lü et al., 2012; *Nesodactylus* from Colbert, 1969). Where necessary data was taken from the radius instead of the ulna. Specimen numbers for *Rhamphorhynchus* are given as in Wellnhofer, 1975. *Additional institutional abbreviations are given in the footnote.

Footnote: Institutional abbreviations for specimens listed in Table 3. AMNH: American Museum of Natural History, New York, U.S.A.; D: Dalian Natural History Museum, Dalian, China; MB: Museum für Naturkunde der Humboldt Universität (Museum for Natural History, Humboldt Museum), Berlin, Germany; MLU: Martin-Luther-Universität Halle-Wittenberg, Germany; MMK: Universitetecs Mineralogisk-Geologiske Instituter og Mineralogisk Museum (University Mineralogy-Geology Institute and Mineralogy Museum), Copenhagen, Denmark; SMD: Staatliches Museum für Mineralogie und Geologie, Dresden (State Museum for Mineralogy and Geology), Germany; TM: Teyler Museum, Haarlem, Netherlands; UB: Katedra geologie a paleontologie Brno (Geological and Palaeontological collection, University of Brno), Czech Republi; UUPM: Palaeontological Museum, University of Uppsala, Sweden.

Several other rhamphorhynchine taxa are known from little or incomplete material making comparisons difficult, but some clear differences are present between all described rhamphorhynchines and *Bellubrunnus*. The recently described *Sericipterus* [2] has a scapula and coracoid subequal in length and highly expanded ends to the proximal and distal ends the first two wing phalanges, neither of which is seen in *Bellubrunnus*, and the anterior teeth are longer with respect to the mandibular length in *Sericipterus* than those of *Bellubrunnus*. The Cuban *Cacibupteryx* [33] has a broader skull with respect to its length than that of *Bellubrunnus*. Similarly the American *Harpactoganthus* [34] has a laterally broad premaxilla and an unusual undulating edge to the toothed parts of the jaws not seen in *Bellubrunnus*. The holotype of *Rhamphocephalus* from the UK is a single large and robust dentary. However, this has sockets for eight teeth which is more than *Bellubrunnus* (with the dentary likely having only five teeth in each ramus) and is very deep anteriorly in *Rhamphocephalus* which is not seen here. Finally the recently described *Qinglongopterus* [35] from China is based on a young animal, although the holotype and only known specimen is split between a plate and counterplate. This new genus differs from *Bellubrunnus* in the presence of the expansion on the distal end of the pteroid in *Qinlongopterus* and some skeletal proportions differ between the two (see Table 3) most notably the ratio of the humerus and metacarpal IV.

### Small or Young Specimens of Rhamphorhynchus

Disagreements remain between taxonomists over the issue of taxonomy *vs* ontogeny for the various putative species named in the genus *Rhamphorhynchus*. Bennett [9] referred all specimens of *Rhamphorhynchus* to one single species - *R. muensteri* - and considered small specimens to represent a series of juveniles that clustered in various size groups as a result of annual sampling, perhaps because of summer storms. In contrast, Wellnhofer [8] favoured a variety of species of different size groups. The smallest of Wellnhofer’s [8] species, *R. ‘longicaudus’*, corresponds with the smallest ‘year class’ (i.e. animals that are around one year old) according to Bennett [9] and these are most similar in size to the holotype of *Bellubrunnus*. However, all of the specimens assigned to the various ‘species’ of *Rhamphorhynchus* fall under the revised diagnosis of Bennett [9]. As *Bellubrunnus* can be distinguished from this definition, it is distinct from all specimens assigned to *Rhamphorhynchus*, regardless of taxonomic regime. A recent study evaluated the year classes of Bennett based on histological sections of bones [36] and while there were some conflicts between these sections and the inferred developmental stages of some individuals by Bennett, those belonging to his year class 1 were all considered very young animals. As such the results of this histological analysis [36] need not be considered further here with regards to the taxonomic issues of comparing *Bellubrunnus* to small specimens of *Rhamphorhynchus*.

Most of the apomophic characters given by Bennett [9] to diagnose *R. muensteri* are present on *Bellubrunnus* indicating a close relationship between the two: jaws with edentulous tips, orbit substantially bigger than the naris and antorbital fenestra [the latter two cannot be observed directly but as is indicated by the size of the sclerotic rings, each orbit is bigger than the entire preorbital part of the skull so this must be correct], the first wing phalanx is the longest and roughly the length of the skull, femur shorter than humerus, prepubis slender and arched with a lateral process.

One character proposed by Bennett [9] to diagnose *R. muensteri* is certainly absent in *Bellubrunnus* however - 34 teeth (four in each premaxilla, six in each maxilla and seven in each dentary) - which Bennett noted was consistent throughout ontogeny in *R. munesteri* (i.e. it is present in all *Rhamphorhynchus* specimens). While some teeth may be missing or hidden under skull elements, with only 21 visible here in the specimen, (and likely only 2 or 3 in each premaxilla), *Bellubrunnus* has a much lower tooth count and a different distribution of teeth in the jaws. It is improbable that some 13 teeth are missing or hidden.

The proportions of major elements and groups (Table 3) are generally similar between *Bellubrunnus* and *R. muensteri*. However, the humerus/femur and ulna/tibia ratios stand out as different for *Bellubrunnus*, especially when compared to the smallest specimens of *Rhamphorhynchus*. The ulna/tibia ratio of *Bellubrunnus* (1.80) is larger than that of any specimen except one large one of *Rhamphorhynchus* of where the ratio is 1.86 for an animal with a humeral length of 19 mm (compared to just 14 mm in *Bellubrunnus*). In the case of the humerus/femur ratio there appears to be a progressive size change with generally bigger specimens having a lower ratio. However, *Bellubrunnus* has a higher value than all other specimens in Table 3, and it is quite different to smaller specimens of *Rhamphorhynchus* despite a comparable size of humeral lengths (13.5–16.5 mm). Collectively these ratios should be treated tentatively given the range of variation seen, but nevertheless, *Bellubrunnus* does seem to have some small differences compared to specimens of *Rhamphorhynchus*, especially those of comparable, or even smaller size, suggesting ontogeny is only a minor issue here.

Wellnhofer [8] used the wing finger being less than ten times the length of the humerus as a defining character of his *R. ‘longicaudus’* though Bennett [9] noted that the transition of this ratio (from less than ten, to ten in larger specimens of *Rhamphorhynchus*) was part of an ontogenetic sequence. While *Bellubrunnus* has a ratio of less than 10, the difference is considerable (the wing finger totalling just 93 mm, with a 14 mm long humerus for a ratio of 6.6) and is lower than all other specimens of *Rhamphorhynchus*. Other differences between *Rhamphorhynchus* and *Bellubrunnus* are the lack of curvature in the distal part of the humeral shaft in *Bellubrunnus*, and the lack of a femoral caput (seen in some specimens of *Rhamphorhynchus*, but not the smallest forms [8]). The morphology of the tail (see below) is also clearly distinct between *Bellubrunnus* and all specimens of *Rhamphorhynchus*.

Collectively then, with the exception of the tooth count and tail morphology, *Bellubrunnus* differs relatively little from *Rhamphorhynchus* compared to other rhamphorhynchines. However, there are differences in various proportions and morphology of the elements that mark *Bellubrunnus* out as different, especially when compared to the smallest specimens of *Rhamphorhynchus*.

### The Tail

The lack of elongate pre- and postzygapophyses on the tail of *Bellubrunnus* and the almost complete lack of chevrons (Figure 9) is clearly unusual for a rhamphorhynchine [26], [29], [30] and worthy of special comment. The general lack of greatly elongated zygopophyses is considered a genuine absence and not though lack of ossification, lack of preservation, or preparation artefact.

The presence of very small and often poorly ossified elements in *Bellubrunnus* such as the palate, gastralia, tarsals, sternum and even the terminal caudal vertebrae strongly suggest that any chevrons and the zygapophyseal expansions would be ossified as well. Chevrons are quite clearly present even in the smallest and less well-preserved specimens of *Rhamphorhynchus* such as the small specimen BSP 1889 IX 1, so their near total absence here and their relatively short form is clearly different. Similarly, in other taxa with similar tails (e.g. *Dorygnathus*) the zygapophyses do not being to taper until some distance after the overlap with the next vertebral centrum in the series. In *Bellubrunnus*, they terminate at the intervertebral articulation with no trace of any extension, and taper quickly to a point suggesting a genuinely different morphology. The general articulation of the specimen and the tail in particular also suggests that these could not have been lost without disturbing the rest of the material, especially as elongate chevrons and zygapophyses would be bound together meaning whole segments of the tails should move or be lost together if these were to be somehow shed.

Finally, a loss by preparation can be positively excluded given the excellent nature of the preparation on the specimen and the fact that at least some are retained. Accidental removal of such tiny structures as the chevrons is not impossible, but as noted above, it seems unlikely that every chevron or zygopophysis could accidentally be removed or damaged without disturbing the rest of the specimen or leaving a trace on the matrix. This morphology then does represent a clear distinction for *Bellubrunnus* compared to other rhamphorhynchine pterosaurs.

### Curved Wing Phalanges

Curved distal wing finger phalanges are known in several pterosaur specimens, both with curvature more mild, and more extreme, than seen here. For example in one specimen of *Rhamphorhynchus* [37] there is a near 90° bend in the phalanx. Others are represented in the literature (e.g. [24], [25], [38]) and further specimens can be seen in some museum collections (DWEH pers obs). However, these are generally isolated cases and such curvature where seen is normally present only on a single wing and is limited to the tip of the phalanx, or the bend is localized at one point on the bone. Thus these examples from other specimens are most likely a result of developmental problems, or the result of stresses on the distal wing during flight causing stress fractures or remodelling.

Here in *Bellubrunnus* however, the distal wing finger phalanges of both wings are curved to an identical degree and the curvature occurs along their entire length of the distal phalanges (Figures 1, 12). More significantly, all other known examples of curvature in pterosaur distal wing phalanges are towards the trailing edge of the wing, they curve posteriorly, whereas here the curvature is anteriorly directed towards the leading edge of the wing. Although elements of both wing fingers have rotated along their long axes (see above), this can be clearly identified as an anterior curvature. All pterosaur wing phalanges have a posterior expansion at their joints that clearly denotes the anterior and posterior faces of the bone and thus shows that the curvature is anteriorly directed (Figure 12). While this is an unusual feature, and may therefore represent a significant diagnostic characteristic, we refrain from considering this an autapomorphy without a second specimen exhibiting the same condition. The curve is considered natural given the lack of distortion anywhere else in the specimen and not a result of stress or a major developmental flaw in the wing given that both phalanges are curved to an equal degree.

**Figure 14:**
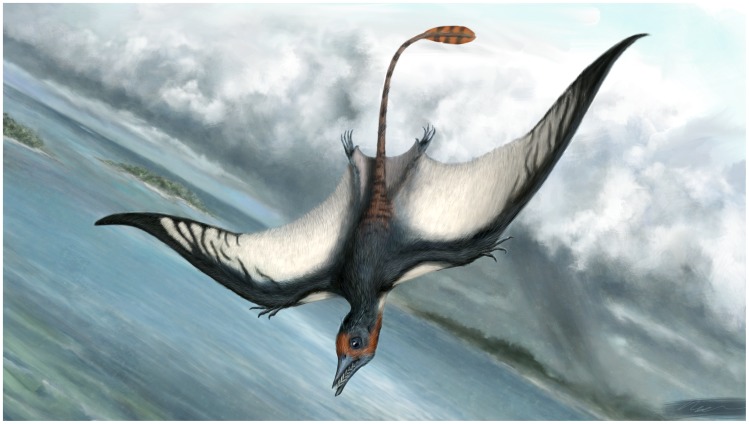
Life reconstruction of *Bellubrunnus*. Image created by Matt Van Rooijen. *Full page width*.

While the curvature is only gentle, this would provide a different wingshape to any other known pterosaur (life reconstruction in Figure 14). Quite what effect this would have had on the wings is not clear, though this likely introduced instability into the wing and allowing for greater maneuverability. The soft tissue component of pterosaur wings had a slightly expanded tip [39] and these tips were densely packed with stiffening actinofibrils [40]. It seems likely therefore than *Bellubrunnus* would have also had unusually shaped wingtips perhaps with a different orientation or arrangement of actinofibrils to avoid wing-tip flutter.

### The Brunn Biota

The fauna and flora of the Brunn lagerstätte are different to that of the Solnhofen type Lagerstätten itself [18]. A flora and fauna similar to that of the Brunn lagerstätte is currently only known from Cerin (Late Kimmeridgian) in France [15]. Numerous terrestrial plants, including conifers, seed ferns, bennetitales and cycadales have been swept in from nearby islands. One of the peculiarities of Brunn is the presence of dasycladacean algae, which are also reported from Cerin. The Brunn fauna is rich in marine invertebrates (e.g. ammonites, brachiopods, echinoids) and the crustacean fauna is dominated by malacostracans and cirripedians. Vertebrates are rare in general, though of the vertebrates recovered so far, fish are by far the most common. Reptiles are extremely scarce with to date only one juvenile turtle, four terrestrial rhynchocephalian specimens [15] and *Bellubrunnus* being known.

Despite the faunal and floral differences to the Solnhofen [18], Brunn is relatively close in time and is effectively identical in palaeogeographical location to the Solnhofen beds. Moreover, the two systems represented at Brunn and Solnhofen do show some similarities in their ecology because both seem to represent coastal lagoonal/reef systems and preserve both marine and terrestrial taxa. Not least the presence of a *Rhamphorhynchus*-like rhamphorhynchid in the Brunn regime suggests an obvious link to the younger Solnhofen regime. Despite the key diagnostic differences shown here between *Rhamphorhynchus* and *Bellubrunnus*, the two taxa are likely to be very closely related and we cannot exclude the possibility that they may represent chronogenera, with the latter transforming into the former. Such a hypothesis is currently untestable, though if *Rhamphorhynchus* is ever recovered at Brunn (or *Bellubrunnus* in the Solnhofen), this would suggest the two were descended from a common ancestor.

It was recently suggested that the extreme similarity of *Qinlongopterus* and *Rhamphorhynchus* indicates a long-term stability of the anatomy of rhamphorhynchines [35]. However, as with *Bellubrunnus*, the holotype of *Qinlongopterus* is known from a single and very young animal, though in rather poor condition. We suggest therefore that the apparent close similarity between the former two genera be most likely attributed to a lack of detailed anatomical information available for *Qinlongopterus*, and the phenomenon of young taxa of different species being more morphologically conservative and resembling one another more closely than do the adults [41].

The broad similarities between Brunn and the Solnhofen in terms of ecosystems and environments, give rise to the possibility that many more pterosaur specimens, and perhaps many more taxa will eventually be found at Brunn. The Solnhofen remains a very important pterosaur fauna and, given the relative lack of new taxa discovered over many years, is likely either complete or close to complete (although material perhaps representing new species remains in private hands - cf. [6] and H.T., E.F. pers. obs.). While the Solnhofen limestone has been quarried extensively, a putative new fauna from Brunn area has, so far, barely been investigated and therefore further excavation may yield more pterosaur specimens that could provide great insight into pterosaur evolution over a short time period, both in terms of individual lineages but also as a complete fauna. The Late Jurassic is an important time in pterosaur evolutionary history as the pterodactyloids first appear and diversify while the basal forms went extinct at the start of the Cretaceous [11]. Thus the potential for two closely allied faunas from this period would be most informative. So far specimens of amniotes are rare from Brunn, though the site has yet to undergo serious scientific excavation. Yet Brunn has already yielded numerous high-quality fossils and we suggest it more likely than not that further new pterosaur specimens and new taxa will be recovered.
